# A pilot mixed methods randomized control trial investigating the feasibility and acceptability of a culturally tailored intervention focused on beliefs, mistrust and race-congruent peer support for Black adults with diabetes

**DOI:** 10.3389/fpubh.2025.1474027

**Published:** 2025-02-06

**Authors:** Meng-Jung Wen, Martha Maurer, Annika L. Pickard, Makenzie Hansen, Olayinka O. Shiyanbola

**Affiliations:** ^1^Division of Social and Administrative Sciences in Pharmacy, School of Pharmacy, University of Wisconsin–Madison, Madison, WI, United States; ^2^Sonderegger Research Center for Improved Medication Outcomes, School of Pharmacy, University of Wisconsin–Madison, Madison, WI, United States; ^3^Department of Clinical Pharmacy, University of Michigan College of Pharmacy, Ann Arbor, MI, United States

**Keywords:** diabetes mellitus, Black or African American, self-management, peer support, health inequities, feasibility studies, mixed methods research

## Abstract

**Introduction:**

Black adults disproportionately experience poor glycemic control and medication nonadherence, yet few diabetes self-management programs address their unique health beliefs, provider mistrust and sociocultural barriers to taking diabetes medications. This 6-month pilot randomized feasibility trial compared a culturally tailored diabetes self-management program, incorporating beliefs about diabetes, mistrust, and race-congruent peer support to a standard diabetes program.

**Methods:**

An embedded mixed methods design examined the feasibility of the pilot trial, including recruitment, retention, intervention adherence and participant acceptability. Data were collected through participant self-reported questionnaires, field notes, semi-structured interviews, and focus groups. Qualitative content analysis inductively explored participants’ feedback on the program, participation barriers and potential strategies to overcome the challenges. Mixed methods integration was implemented using a side-by-side joint display to compare, synthesize and interconnect the quantitative and qualitative results across all feasibility domains.

**Results:**

Thirteen participants (93%) completed the trial, demonstrating high adherence and retention. Community outreach and a prerequisite orientation using motivational interviewing were feasible and appropriate to recruit potential participants. Participants expressed high satisfaction and acceptability, highlighting the importance of peer support, cultural relevant content and a safe space for sharing experiences. Barriers to participation were identified including schedule conflicts and difficulties in engagement.

**Discussion:**

Future large-scale effectiveness trials should consider combining multimedia into recruitment methods, tailoring the program to address medication-taking goals, and addressing social and environmental barriers to support sustained lifestyle changes.

## 1 Introduction

Black adults face significantly higher rates of type 2 diabetes-related complications and mortality compared to non-Hispanic white adults (1–4). One factor accounting for these disparities among racial and ethnic groups is the higher rates of diabetes medication non-adherence among Black adults compared to white adults (5–9). Medication non-adherence is associated with higher hemoglobin A1C levels, which in turn exacerbates diabetes-related health complications (10–12). Prior research identified concerns about medicines, misperceptions about the necessity of medicines and provider distrust to be contributing factors to medication non-adherence among Black adults (13–15).

Despite the breadth of topics that diabetes self-management programs address, some critical content is missing. Diabetes self-management programs typically include content about common diabetes health concerns (e.g., nutrition, foot exams), but for Black adults, these programs have not demonstrated a sustained effect on improving A1C (16, 17). These programs do not adequately address medication adherence (17), and do not modify their content to be relevant for Black adult’s psychosocial and sociocultural concerns contributing to diabetes disparities (18), including medication self-efficacy, health misperceptions (19), and experiences of racial discrimination/distrust in the context of healthcare (16). A study by Andreae et al. tested an intervention using storytelling to address diabetes medication adherence for a sample of primarily Black adults. While medication adherence was improved, the intervention had no effect on A1C and notably did not address communication among patients and providers (20).

To address the limitations of existing diabetes self-management programs, we developed an intervention that focuses on addressing Black adults’ perceptions of diabetes and medicines as well as distrust in providers (21–23). This intervention provides Black adults with information about diabetes and medication beliefs to address misperceptions, develops behavioral skills by augmenting self-efficacy/self-advocacy to enhance communication with providers, and provides one-on-one race-congruent peer support toward improving medication adherence. Our prior research has demonstrated that race-congruent Black adult peers with diabetes can provide culturally appropriate informational support and increase engagement in medication adherence in ways that clinicians cannot through clinic visits alone (22, 23). Peer support can enhance self-advocacy toward communicating with providers (24).

Engaging Black adults in research is challenging at the stage of recruitment as well as when supporting their ongoing participation throughout the study. Black adults are underrepresented in health research and historically have had low participation rates in clinical trials (25). Low Black adult participation in research has been attributed to several factors: (1) fear and distrust of medical or University-research and healthcare systems due to awareness of past unethical research practices and equating medical research with negative healthcare system experiences (26–30), (2) concerns about sharing personal information and confidentiality (27, 30), (3) competing priorities and logistics such as lack of time, transportation, child care (27), (4) inflexible research protocols, which do not allow for recruiters to establish a connection with participants (27), (5) recruiters who are a different race or ethnicity than the potential participant (31), (6) beliefs that research will not benefit them individually or their own communities (32), and (7) concern with randomized controlled trial design that the participant will not receive the beneficial treatment (32). Given the disproportionate number of Black adults who are diagnosed with diabetes and experience related morbidity and mortality, there is a critical need to address barriers to participation to facilitate increased enrollment. To successfully implement and sustain evidence-based interventions in the real-world setting, it is essential to assess the feasibility of recruitment methods, fidelity of intervention delivery and participant’s acceptability of the intervention components in a pilot trial. This study aimed to assess the feasibility and acceptability of a culturally tailored diabetes self-management program. Specifically, we assessed the feasibility of recruitment methods, the fidelity of intervention delivery, and participants’ acceptability of the intervention components through a mixed methods randomized controlled pilot trial. The trial results regarding the clinical and psychosocial outcomes will be published elsewhere.

## 2 Methods

### 2.1 Ethical statement

This randomized controlled pilot feasibility trial was approved by the Health Sciences Institutional Review Board of the Principal Investigator’s University (STUDY ID: 2020–1061). Participant data were de-identified and stored securely on password-protected computers in a locked office. Detailed protocols ensured compliance with institutional and legal standards for confidentiality a written informed consent form was obtained from each participant prior to the trial. No personal identifiers of participants were retained after data transcription. The clinical trial is registered at: https://clinicaltrials.gov/ct2/show/NCT05527847.

### 2.2 Research design

A pilot randomized control feasibility trial examined the study’s feasibility and participant acceptability. This study used an embedded mixed methods design to integrate qualitative and quantitative data, focusing on feasibility and acceptability. The protocol of this trial has been published previously (33). The study was conducted in one community location in a Mid-Western State from February to November 2023.

### 2.3 Participants

Fourteen participants were enrolled in the trial. The eligibility criteria were (1) adults aged 18–90 years old, (2) diagnosed with type 2 diabetes for at least 1 year, (3) self-identified as African American/Black, (4) can speak and read English, (5) self-reported being prescribed a diabetes medication, (6) self-reported nonadherence on the 3-item Domains of Subjective Extent (DOSE) of Nonadherence survey, and had an (7) A1C value greater than 7.5%. Participants who self-reported bipolar or personality disorders, schizophrenia, Alcohol and Other Drug Abuse (AODA), dementia, had a history of severe hypoglycemia requiring medical assistance or glucagon administration, and reported participating in another lifestyle or medication adherence program were excluded.

### 2.4 Recruitment and randomization

Participants were recruited actively through community partners network, in-person community events, churches, and senior centers. We also used passive recruitment methods such as posting flyers in the community location, advertisements in a newspaper commonly read by African American/Black audiences, websites of a local senior center and promoting the program through radio interview. People who were interested in participating contacted the study coordinator via phone to screen for eligibility or directly fill out the screening questions via a QR code linked to REDCap.

After completing the screening questions, the study coordinator followed up with potential participants to schedule an in-person screening with the research team. During the screening day, the research team members collected Hemoglobin A1c at point of care to assess eligibility and conduct a 30-min prerequisite orientation. This prerequisite orientation adapted a methods-motivational interviewing approach and occurred before obtaining participants’ informed consent (34). The research team explained the study information and all study expectations to potential participants and left time for a small group discussion to share concerns about trial participation. After receiving written informed consent from all participants, participants were randomized into the intervention (Peers EXCEL) group and the control (Health Living with Diabetes, HLWD) group.

### 2.5 Intervention

#### 2.5.1 Intervention group (Peers EXCEL)

Peers EXCEL was an 8-week culturally tailored program for Black/African American adults with uncontrolled type 2 diabetes, incorporating group education sessions addressing provider mistrust, beliefs about diabetes, race-congruent peer phone call support and community health worker support for issues related to social determinant of health including housing, food insecurity, transportation, etc. Previously, we developed Peers EXCEL by adding Peers LEAD, a culturally tailored program for African Americans with type 2 diabetes to Healthy Living with Diabetes (HLWD), a widely disseminated, evidence-based diabetes self-management program in Wisconsin (35). The details of the effectiveness of the HLWD program in improving patient outcomes, as well as utilization and cost outcomes, can be found in the next section for the control group. The development and pilot of Peers EXCEL and how it showed a clinically meaningful decrease (−0.7%) in mean hemoglobin A1C, a signal of improving medication adherence and other psychosocial outcomes, has been published elsewhere (22).

**Figure 1:**
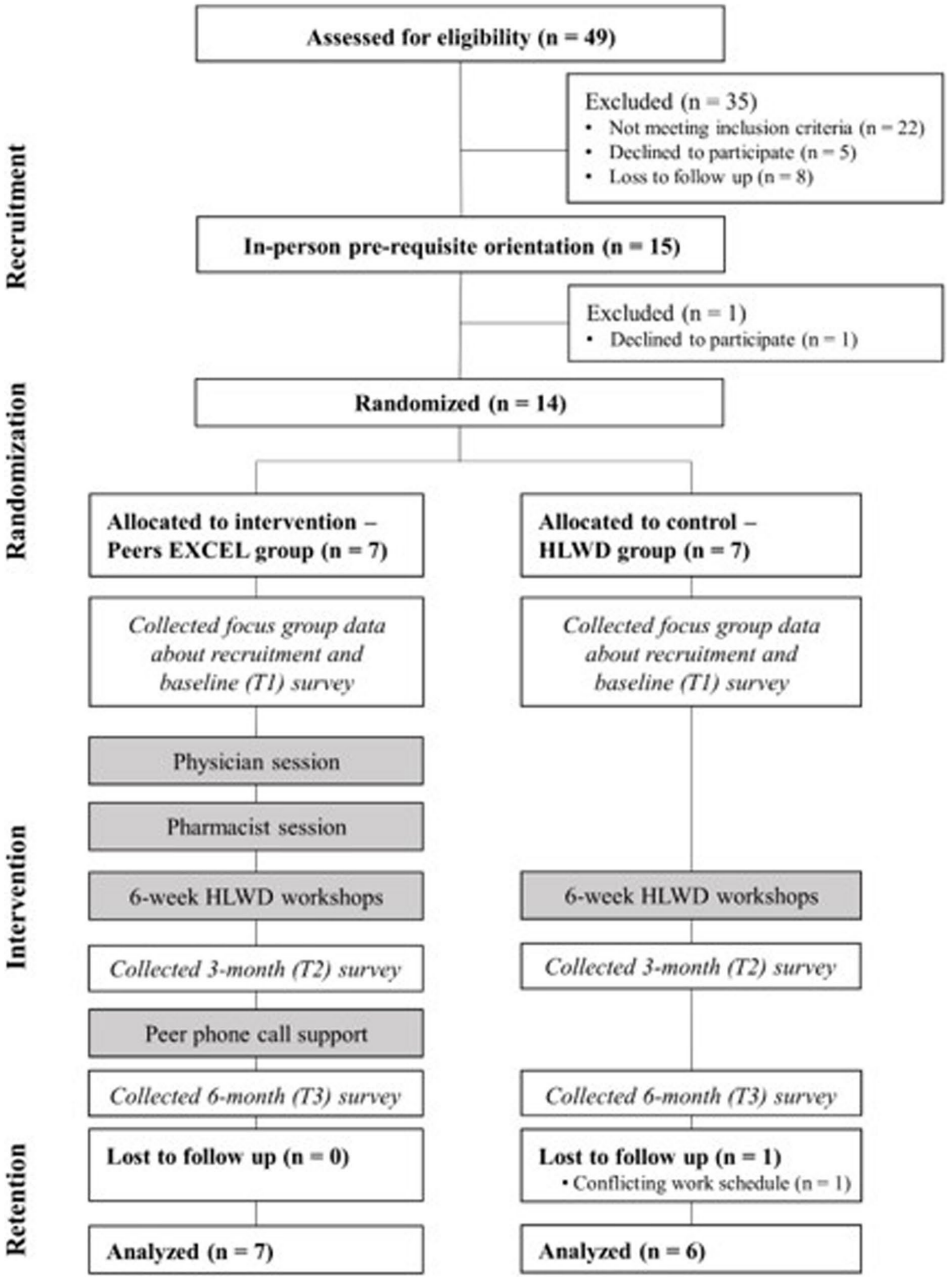
Consolidated Standards of Reporting Trials (CONSORT) diagram of participants in each process and each assessment time point of the trial.

The 8-week intervention consisted of three components: (1) eight weekly 2.5-h, in-person group sessions – including two provider sessions and six HLWD self-management workshops, (2) referral to community health worker if requested for addressing the need of social determinants of health, and (3) regular peer-based phone call support with an ambassador – a Black peer with controlled type 2 diabetes (as determined by a recent A1c value less than 7.5%). The first provider session led by a physician covered addressing provider mistrust and initiating a conversation with healthcare providers. The pharmacist session followed the physician session and involved a discussion about beliefs about diabetes and addressing misbeliefs about diabetes medications. The subsequent six-week self-management sessions were led by two Black HLWD-trained facilitators, focusing on topics related to diabetes self-management – diet control, exercise, and stress management. The Consolidated Standards of Reporting Trials (CONSORT) diagram in Figure 1 further described the flow of participant through study at each time point.

#### 2.5.2 Control group (HLWD)

Participants in the control group received HLWD, a standardized, evidence-based diabetes self-management program, which consisted of six workshops covering topics related to diabetes self-management. The program has demonstrated evidence of improving glycemic control and psychosocial outcomes, such as enhanced self-efficacy, and reduced diabetes distress. It has also led to a ~ 53% reduction in emergency room visits, resulting in lower healthcare expenditures (35). Participants also received support from the community health worker, if requested.

### 2.6 Data collection

#### 2.6.1 Quantitative measures

Quantitative data including study coordination documents and self-reported questionnaire were collected for assessing trial feasibility and acceptability. Feasibility in this study refers to the extent to which the intervention can be successfully implemented in a community setting. We evaluated trial feasibility by assessing four key areas: (1) recruitment and retention capability, (2) data collection procedures, (3) intervention adherence, and (4) intervention acceptability. Acceptability, in this study’s context is defined as participants’ perception of the intervention as agreeable, palatable, or satisfactory (36). For recruitment and retention capability, one study coordinator documented recruitment method, dates of recruitment and enrollment and dropout information throughout the program. For intervention adherence, we collected group attendance sheets and peer phone call completion forms. All forms were documented through REDCap platform, a secure data management platform, by the research team members.

At the end of the intervention (6-month timepoint), a 20-min self-reported survey collected in-person, was used to understand participants’ feedback about the intervention. For data collection adherence and acceptability, four survey questions, using a 5-point Likert scale, from ‘very burdensome to very comfortable,’ included participants’ perceptions and comfort levels regarding the data collection process. We added one question, with three options to assess participants’ perceptions of the time to complete the survey, including ‘less than I expected,’ ‘about what I expected,’ and ‘more than I expected.’ To assess participants’ acceptability of the program and satisfaction ratings, we used two validated questionnaires measuring implementation outcomes – Acceptability of Intervention Measure (AIM) and Intervention Appropriateness Measure (IAM) (37). The two measures, each with four items, used a five-point Likert scale – from ‘completely disagree’ to ‘completely agree’ to evaluate trial participants’ perceptions of the acceptability and appropriateness participating in the intervention. We also used a five-point Likert scale, from ‘not at all useful’ to ‘extremely useful,’ with seven questions asking participants to rate the usefulness of each intervention component in the trial.

#### 2.6.2 Qualitative data

We conducted two focus groups with participants in the intervention and control groups to explore their feedback on the study recruitment strategies, including recruitment locations, materials, and the prerequisite orientation which occurred before informed consent. The two 30-min focus groups were conducted by a research scientist, who has extensive qualitative training and has experience in facilitating focus groups.

Semi-structured in-person participant interviews were conducted by research team members at two time points, including after the group sessions lasted and at the end of the 6-month trial. Each interview lasted between 30 and 40 min. Questions included in the interview guide covered participants’ feedback on weekly group sessions, peer support phone calls (intervention only), the data collection process, and their perceptions on how they incorporated diabetes knowledge and self-management skills into their daily lives. As well, to monitor retention of participants, documented study coordination field notes were used to evaluate why participants decided to drop out throughout the trial.

### 2.7 Data analysis

#### 2.7.1 Quantitative data

Descriptive statistics were used to analyze the feasibility of implementing the pilot trial with IBM SPSS Statistics (Version 28.0). The research team calculated the total enrollment rates, the duration of recruitment and the proportion of participants recruited from each recruitment method. The group session attendance rates, participant dropout rate and retention rate was collected. For participants in the intervention group, the research team recorded the completion rate for phone calls between participants and their ambassadors during the 6-month trial.

#### 2.7.2 Qualitative data

Participants were selected through purposive sampling recruited through various methods such as face-to-face, radio and word-of-mouth. The two focus groups, and individual semi-structured interviews were audio recorded and analyzed using NVivo version 10. We completed inductive content analysis (38). The data was initially coded line by line by research team members with training in qualitative data analysis. Qualitative content analysis included developing codes, themes, and subthemes. These themes contained subthemes and codes which have corresponding language, and interconnectedness. In this study we established rigor and trustworthiness using four criteria created by Lincoln and Guba (39). These criteria include transferability, confirmability, dependability, and credibility.

#### 2.7.3 Mixed methods integration

Using a mixed methods intervention design, we compared, synthesized, and interconnected the quantitative and qualitative results across four feasibility domains. Integrating and merging quantitative and qualitative data using a mixed methods design augments a comprehensive understanding of the feasibility and acceptability of conducting a behavioral trial. The qualitative data collected throughout the study was used to explain the results of the quantitative measures in each trial feasibility domain and provide implications for future fully powered trials. In this study, we followed Aschbrenner et al.’s principles and considerations when planning for the pilot trial, aiming to explore the feasibility and accessibility of trial implementation and implications for a future fully powered randomized controlled efficacy trial (40). Our mixed methods research questions were developed based on three reasons for integration – (1) triangulation, which involves comparing quantitative and qualitative data to confirm or contrast key elements of feasibility; (2) completeness, which entails generating new evidence about feasibility by synthesizing quantitative and qualitative data that examines different aspects of the trial implementation; and (3) explanation, which aims to clarify differences in feasibility among subgroups by linking quantitative and qualitative data (40). The corresponding mixed methods questions and the reasons of their integration were provided in Supplementary material 1. The four key feasibility domains: (1) recruitment and retention capability, (2) data collection acceptability, (3) intervention adherence, and (4) intervention acceptability, were investigated to understand facilitators, barriers and strategies for improvement.

Integration was also implemented at a methods level. To assess recruitment and retention capability, we triangulated the recruitment rate by recruitment sources and participants’ feedback to identify the most feasible and effective way to recruit participants. We synthesized the enrollment and retention rates with qualitative information to identify recruitment and retention facilitators and barriers as well as future directions toward improving the study process. Qualitative data regarding participants’ experiences conducting the survey, interview and focus group expanded our understanding of participants’ satisfaction ratings regarding the data collection process. Areas for modifying the implementation of the intervention were identified.

In the feasibility domains of intervention acceptability and adherence, we compared and synthesized participants’ quantitative measures (e.g., intervention acceptability ratings and attendance data) with their experiences with each intervention component (i.e., group sessions, peer phone call support, community health worker support). To develop strategies to enhance intervention adherence, we compared the differences in qualitative findings by interconnecting subgroups with high/low intervention adherence rates. For each sub-group of high versus low intervention adherers, we identified facilitators and barriers to adhering to the intervention as well as how diabetes self-management practices were incorporated into their daily life. We used a data collection joint display to detail the types of quantitative measures and qualitative data collected at three time points (baseline, after the group sessions and at the end of the program), research questions and meta-inferences for four feasibility domains. This is included in Supplementary material 1.

## 3 Results

### 3.1 Participant demographics

Fourteen individuals participated in the trial, and 13 finished the 6-month program. One individual from the control group withdrew due to a schedule conflict. On average, the participants were 54.6 years old (standard deviation = 17.4), with seven (54%) identifying as female. The average duration since diabetes diagnosis was 14.7 years (standard deviation = 9.6), and they typically took 1.9 (standard deviation = 1.2) diabetes medications.

### 3.2 Quantitative trial feasibility outcomes

#### 3.2.1 Recruitment and retention capability

Our recruitment methods reached 49 (102% of our target) potential interested participants, exceeding the initial target of 48 people we expected to express interest in participating.

Two-thirds of participants were actively recruited in person (*n* = 23, 69%), primarily through family and friends’ word of mouth (*n* = 12, 36%), faith-based organization events (*n* = 8, 24%), or clinic referrals (*n* = 3, 9%). One-third of participants (*n* = 10, 31%) were recruited via passive channels, such as online newsletters or advertisements (*n* = 4, 12%), flyers (*n* = 3, 9%), or radio (*n* = 3, 9%). Our initial recruitment goal was to enroll 24 participants; however, our recruitment methods were only able to reach 15 participants (63% of our target) who were among those eligible for the study. The main reasons for ineligibility were A1C < 7.5% (*n* = 12), followed by not being diagnosed with type 2 diabetes (*n* = 5). Eight potential participants could not be reached for follow-up during the recruitment stage.

Although we did not meet our targeted recruitment goal, we exceeded our revised goal for recruiting and enrolling participants. The trial enrollment rate was 93% (14/15, higher than the 80% target). The enrollment duration was 13 days, which was 47 days faster than we anticipated. No participants dropped out after knowing the randomization results (20% lower than our target). The retention rate was 93% at the end of group education sessions (100% in the intervention and 86% in the control). The overall retention rate remained at 93% at the end of the 6-month program. The reason for one participant dropping out was due to conflicts with their work schedule.

#### 3.2.2 Data collection acceptability

The feasibility process for data collection seemed to be acceptable among participants. Based on survey self-reported responses, participants reported that collecting A1C, blood pressure and survey data was comfortable (mean = 1, SD = 0.9). The time for completing the survey was as expected (mean = 2, SD = 0.6) and the interview took less time than participants expected (mean = 1, SD = 0.9).

#### 3.2.3 Intervention adherence

High rates of intervention adherence were reported in the trial. Participants average group session attendance met our target of 80%. We also categorized participants into two groups based on their intervention adherence. Nine participants had high intervention adherence (5/7 in the intervention group and 4/6 in the control group). Those who did not meet our target were categorized as lower intervention adherence group (*n* = 4, 2/7 in the intervention group and 2/6 in the control group). Additionally, in self-reporting their diabetes self-management tasks, participants with low adherence had low confidence levels in maintaining a regular healthy diet (mean = 5.8, SD = 12) after the group sessions. For the peer phone call support in the intervention group, the mean completion rate of phone calls was 5.6 (SD = 2.5).

#### 3.2.4 Intervention acceptability

Participants reported a high level of acceptability regarding the program. The overall satisfaction rating was ‘satisfied to very satisfied’ (mean = 4.75, SD = 0.3) and the quality of the HLWD workshop was rated as ‘good to excellent’ (mean = 3.8, SD = 0.3). Participants expressed ‘very satisfied’ (mean = 5.0, SD = 0.0) with the group leaders, while the workshop time received the lowest satisfaction rating among intervention (mean = 4.45, SD = 0.7) and control groups (mean = 4.6, SD = 0.6). Participants found the program to be ‘acceptable to very acceptable’ (4.6, SD = 0.7) and felt it was ‘appropriate to very appropriate’ (4.6, SD = 0.9) for Black adults with diabetes. Related to the intervention components, the pharmacist session (4.8, SD = 0.4) and peer phone calls (4.7, SD = 0.8) were rated as the most useful with the community health worker support considered the least useful (4.2, SD = 1.3).

### 3.3 Qualitative findings of trial feasibility

#### 3.3.1 Recruitment and retention capability

##### 3.3.1.1 Reasons for participating in the program

Participants in the program reflected on their reasons for participation and reported the benefits of receiving more knowledge about diabetes and its severity, continuous support from peer individuals with diabetes and motivation to be an advocate for people in similar situations.

“I just wanted to have more resources, more information and be more educated for myself and with others that’s around me, so I’m able to talk with other people and tell them about these things. I’m being an advocate for other people who are struggling much like I was when I was first diagnosed.” (P3, control group).

##### 3.3.1.2 Facilitators that enhanced participant recruitment

Almost all participants preferred to have more interactive approaches for recruitment. They liked how community members and study members showed up in community events to reach out to potential participants in person. Introducing study information and being ready to answer questions in real time were reported to be the best ways to recruit participants.

“[PI] was at the pantry, St. Vincent’s…she was there… And that was nice to see someone coming to me instead of…I read it in the paper, and I have to make the phone call. It was nice that somebody was there.” (P4, intervention group).

Before consenting to participate in the program, we held a prerequisite orientation for all potential participants to provide introductory information about research, study information, timeline and expectations. Participants reacted positively to this session, noting that it gave them an opportunity to learn about the program and research principles such as randomization. They enjoyed the opportunity to be informed of all the study expectations.

“It was a great introduction to the program, just letting us know what we are getting involved in and what is expected of us and what resources or what information you all would give.” (P3, control group).

##### 3.3.1.3 Longer time commitment and work schedule conflicts were the main barriers to participation

Some participants felt uncertain about the long commitment to a 6-weeks, in-person program. They expressed their worries about being present at 2.5-h weekly group sessions, mainly because of their work schedule. One participant said,

“I have kind of an erratic schedule, so sometimes I’m not sure where I’m going to be. I wasn’t sure if I was going to be able to commit to a certain time every week.” (P1, intervention group).

##### 3.3.1.4 Suggestions for future recruitment

Participants provided several possible solutions for recruitment. They suggested utilizing social media platforms. Specifically, they indicated using short videos of previous program participants sharing their personal stories, so potential participants could resonate with the experiences from others.

“That would be perfect as a recruitment tool… (for example,) social media…if you had a person, video one of our sessions…and then we could, you could throw that out there on the social media… I’d like to get a piece of that.” (P7, intervention group).

The study team experienced a lot of challenges recruiting people who met our inclusion criteria. Some participants thought the eligibility of participating in the program were too strict in this geographic region and suggested to include more people such as family members and friends,

“Sometimes it’s good to have a family member understand what’s going on because that person might be the one that need the help, the person that has diabetes.” (P1, intervention group).

#### 3.3.2 Data collection acceptability

##### 3.3.2.1 Helpful to keep track of hemoglobin A1c levels and blood pressure

Participants appreciated that they received an update on their A1c levels, as it helped them gain understanding of their current health condition. One of the participants stated,

“I think data collection is very helpful. It lets people know where they are at with their A1C, other than waiting three months to go get it checked.” (P5, intervention group).

##### 3.3.2.2 Survey questions are too long and can be refined

Though participants understood the survey material, they reported that there were many questions that took time to complete.

“Survey was clear. It was just probably a little longer than I wanted it to be…Probably did not need as many questions as you did.” (P10, intervention group).

##### 3.3.2.3 Suggested to use electronic methods to collect study data

Participants suggested using electronic methods to collect data instead of using paper copies.

“It’s easier for you to analyze if you have the data electronically. Because, you have to go back, and you have to key it in.” (P1, intervention group).

#### 3.3.3 Intervention adherence

##### 3.3.3.1 Frequency of meeting helped with focus and accountability

Participants generally appreciated the regularity of group sessions and peer ambassador phone calls (only in the intervention group), which kept them focused on diabetes self-management. With weekly group education sessions, they acknowledged the ability to plan for their health goal and make efforts to achieve it.

“We had to set goals, and we had to follow up on our goals and discuss what we did throughout the week. And so that kept, it held me accountable…. Weekly meetings because that, with the group, because that reinforced, again, it reinforced what I needed to do, and it kept me focused. And that type of accountability makes a big difference because it’s so easy to say I’m going to do something but not do it” (P1, intervention group).

##### 3.3.3.2 Challenges with program engagement

Some participants expressed difficulty confiding with other group members during group sessions. During group discussions, participants were encouraged to share their individual experiences with diabetes, so if a participant was not comfortable sharing that information, it could feel like they were being coerced to share. One participant detailed this discomfort,

“Sometimes they wanted you to speak on issues that you did not want to speak on. So they are trying to force the issue or force the issue out of you. And it’s like, nah, I really do not want to comment. Just let me pass.” (P10, intervention group).

Some participants felt the program’s format was not as interactive as it could have been. During weekly sessions, facilitators read off the HLWD script, which did not give the participants much opportunity for participation. One participant said,

“I did not like the instructors [HLWD workshop facilitators] reading word for word on how to do things. I can read myself. I do not need you to read word for word …. There was no real interaction as far as allowing people to get up.” (P4, intervention group).

Participants reported the most common barrier to attending the group sessions was personal or family life events. Many participants had families, jobs, and busy schedules to attend to. If emergencies came up within their family or work, they had to prioritize those events over the program. Participants who had to skip may have missed out on important topics related to their diabetes management and had less engagement with the program. One participant acknowledged what a family emergency meant in regard to the program,

“We had a death in the family, so I had to go out of town. … I was able to Zoom in on that. So that was nice they allowed that for me.” (P4, intervention group).

Regarding the length of program, some participants wanted to have a longer duration while others preferred short period of time for the group sessions because of a busier schedule. One participant suggested the intervention could have been longer to teach the participants even more about diabetes management and further strengthen the sense of community and support within the group.

“What was it, 8 weeks, so maybe if it was 10, 12 weeks, I think it might have been, for me, I think it might have been better because then … I might have been able to kind of continue a little bit more on my own. (P1, intervention group).

Finally, participants felt they lacked the time necessary to read the assigned material. Participants were given a diabetes management book at the beginning of the trial, and each week, the facilitators assigned readings in the book related to session topics. One participant reported the burden of reading such an amount of reading on a weekly basis outside of the work schedule,

“To be honest, it was hard to read so many chapters in a week with life and work and everything else going on.” (P1, intervention group).

##### 3.3.3.3 Maintaining healthy lifestyles in a long term

Regarding long-term behavior maintenance, majority of participants recognized the potential of applying diabetes self-management knowledge and skills they had learned in the program to their daily lives including dietary adjustments, exercise, and medication management. One participant discussed an example of changes,

“I was making a lot of diet decisions that were unhealthy for me prior to entering the program. … as I participated and went along week by week with the program, that diet … grabbed my conscience. It made me conscious and kind of gave me some, a little bit of control. I was able to gain a little bit more control over my diet. And that control is good. Control is paramount.” (P7, intervention group).

However, some participants also mentioned challenges when it came to creating healthy habits and maintaining them. Some perceived that there are barriers to making a permanent change because their old habits could resurface. One participant explained what they thought about breaking these old habits,

“There’s always going to be barriers (of breaking old habits), because it’s hard to break a habit that you have been into for so long. But if you have that mindset that you need to change that barrier, then you change it.” (P5, intervention group).

Participants further indicated there are social and environmental barriers to overcome when creating new habits. Participants reflected that diabetes management often becomes more difficult when there are negative influences from family and friends, such as pressure to eat unhealthy food. They reported the environment could play a role too, especially when their surroundings do not promote physical activity or a healthy lifestyle. One participant mentioned how a factor out of their control impacted their exercise routine and resulted in weight gain,

“That’s my exercise thing, and I walk outside sometimes at the apartment. But lately, they closed the pool from June, and they just opened up yesterday, so hot dog. I gained ten pounds.” (P4, intervention group).

#### 3.3.4 Intervention acceptability

##### 3.3.4.1 Learned and gained resources for coping with diabetes

Participants appreciated the resources provided during the intervention. For example, participants were provided diabetes education materials, which they reported prior non-access despite their lengthy history of diabetes diagnosis. One participant explained the general benefits of participating in the program,

“Having these resources available has been a blessing to me because I’ve learned more about my condition than I have in the past ten years.” (P3, control group).

One participant specifically recalled the coping skills related to healthier lifestyles and emotional control he learned from the program.

“(I have learned) how to manage my food, how to write down how much sugar and stuff in the food, how to handle depression … when you are going through, like you can occupy your mind with music, praying, reading.” (P9, control group).

##### 3.3.4.2 Group-based learning environments motivated learning

Several participants mentioned that the group-based learning approach was helpful for building connections and sharing their personal stories. Participants indicated that learning diabetes-related knowledge with people in similar situations was motivational. As well, they reported opportunities to share their experiences and tips with others when discussing other culturally relevant topics.

“…and then what also made it good was the group feeling that I would have when I came in and I was among the group of other people like me. And so those kinds of things there caused me to be able to learn more. (P7, intervention group).

To enhance participants’ engagement during group sessions, the HLWD workshop facilitators opened up about their own lived experiences to encourage others to join in the discussion,

“I learned more, I learned quite a bit from the two ladies [facilitators], because they also talked about their experiences, which helped a lot. I thought, I’m not in this alone. They had some of the same problems that I did, (for example,) with missing meals.” (P6, control group).

##### 3.3.4.3 The dietary modifications discussed with the facilitators resonated with cultural background and experience

Participants also acknowledged the cultural adaptation of the program content for Black adults with type 2 diabetes. Participants expressed that some of the diabetes topics were tailored to their personal needs because facilitators modified the examples when discussing how to manage their diet.

“They (content in program) were relevant to me because growing up, we prepared our food a certain way with a lot of oils and fats and things like that made everything taste so awesome. But with this program, I learned how to alter the taste by using different seasonings and spices… trying to use alternatives… And I did not miss all the other stuff I used to have. I do not miss it … that’s where culture would come in, culture specific, because in our culture, African Americans, we eat different. … and our ingredients are different.” (P7, intervention group).

##### 3.3.4.4 Suggestions for future program to enhance program acceptability and accessibility

The participants had some concerns regarding the community health worker. They pointed out that the race and age dissimilarity between themselves and the community health worker may have resulted in the CHW lacking understanding of the participant’s lived experiences. One participant said,

“We are all black, and he [community health worker] is white… So he might not have understood. And he was young, and most of us are older. So he might not have understood what we were going through.” (P1, intervention group).

There were several positive comments regarding how the facilitators led each session. Participants felt the facilitators took the time to understand them by being inclusive and welcoming despite their age differences. One participant who noted being the youngest mentioned,

“I did want to say they [the facilitators] were very always upbeat and very genuine people that I really liked to work with. And I know, they were kind of like not young, young, but they were, kind of in the middle, like they can understand me, and then they can understand everybody else, which I really enjoyed too.” (P2, control group).

To enhance program accessibility, some participants suggested having electronic versions and multimedia forms of the educational materials which would encourage participants’ access to the self-management book, discussion highlights and video reminders of the skills they have learned. One participant reported,

“I think one of the things that might have been helpful was, I mean, they gave me the big book, and I did not have a lot of time. So if there was some audio, maybe an audio book, so even links to sites, to videos, to short videos and things like that, … things like that would have been helpful for me.” (P1, intervention group).

Some participants recommended the addition of more information about exercise, health insurance and input of other healthcare professionals such as dietitians into the program. For example, the participants expressed how more session time dedicated to physical activity could be beneficial, with one participant elaborating.

“I think I would have an exercise day, an exercise session, where you’d have somebody in there who can just kind of give a little bit of advice on some different types of exercises you can do, something physical, a physical activity session. I think that’s probably the one thing that was missing from the whole thing [program]. (P1, intervention group).

### 3.4 Mixed methods findings

Quantitative results of feasibility outcomes showed in-person recruitment was the most appropriate way to recruit Black adults with type 2 diabetes into a self-management program. We met our goal for enrollment and retention in the trial. The randomization results and data collection process were acceptable for participants. Participants had high adherence to interventions and reported high satisfaction and acceptability to the program. Participants also perceived the program was appropriate for Black adults with type 2 diabetes and the program contents and support were useful for them. Integrated with corresponding qualitative data, qualitative themes and quotes further confirmed and expanded the trial feasibility outcomes. The joint display in Table 1 shows feasibility outcomes including quantitative results and the qualitative data summary which provided implications for addressing key intervention feasibility questions.

**Table 1:** Mixed methods integration using joint display to examine trial feasibility.

Feasibility domains	Quantitative results	Qualitative data summary	Meta-inference
Recruitment and retention capability	Two-thirds of participants were recruited in person (*n* = 23, 69%)	Appreciated the community-based approach to recruiting potential participants. Preferred interactive ways to learn about the program (e.g., prior participants ‘sharing their experience, PI radio interview).	Confirmed by the quantitative and qualitative data, community outreach was a feasible and effective way for recruitment. An effective way to deliver study information is to utilize community members to share personal experiences with potential participants and build an emotional connection.
	Enrolled rate = 93% (14/15, 13% higher than target) Enrollment duration = 13 days (47 days faster than target)	Positive experiences on the prerequisite orientation due to the clear explanation of research terms, better understanding of the study information and expectations, and providing opportunity for potential participants to raise concerns	Implementing a prerequisite orientation is highly acceptable for participants and may be useful in enhancing enrollment rates.
	Dropped out after randomization = 0% (20% lower than target)	Appreciated the prerequisite orientation, which explained the concept of randomization in a clinical trial and the rationale behind the process. No preference for a specific trial group after learning about the interventions	Implementing a prerequisite orientation may enhance the acceptability of the randomization process and results.
	Retention rate = 93% (13/14, 18% higher than target)	One participant dropped out due to work conflicts.	The main barrier to participating in the program was conflicts with work schedules.
Data collection acceptability	Data collection process was comfortable^a^ for participants (mean = 1.7, SD = 1.2) Time to complete the assessment was as expected (mean = 1.6, SD = 0.7)	Appreciated the opportunity to get updates on A1c and blood pressure values while the negative aspects included too many survey questions and some questions were repetitive. Suggested collecting data electronically.	Refining survey questions, reducing the number of survey items and using electronic methods to collect data may alleviate participants’ burden.
Intervention adherence	Group session attendance = 80% (met our target) Number of completed phone calls = 5.6 (SD = 2.5) out of 7	Weekly group sessions enhanced accountability. Ambassador phone calls served as reminders of prior education, helping participants stay focused. Work schedule conflicts and family emergencies were barriers to participating in the group sessions.	Regular group sessions with peer support motivated participants’ attendance whereas schedule conflicts and family emergencies were the main reasons for missing group sessions.
	Participants with low intervention adherence^b^ (*n* = 4, 31%)	Difficulties in sharing thoughts related to diabetes self-management among other people made low adherence group participation difficult. Finding it hard to break old habits (e.g., smoking) were challenging for the low adherence group in maintaining diabetes self-management practices.	Barriers to engaging in group discussion could be further explored and addressed in the low adherence group. Reframing medication beliefs and addressing social barriers in the low adherence group could be helpful in incorporating self-management practices into daily life.
Intervention acceptability	High satisfaction^c^ (mean = 4.75, SD = 0.3) High acceptability^d^ (mean = 4.6, SD = 0.7) High appropriateness^d^ for Black adults with diabetes (mean = 4.6, SD = 0.9)	Liked the resources, education, and safe space provided to share. The interactive nature of the education received enhanced their self-efficacy for diabetes management. Dietary modifications discussed resonated with their cultural background and experience.	Confirmed by quantitative and qualitative data, the program was highly acceptable and appropriate for Black adults with type 2 diabetes.
	CHW support was least useful^e^ (4.2, SD = 1.3) among intervention components.	Fewer opportunities to interact with CHW and preferred individuals with similar backgrounds and lived experiences.	Having race-congruent support from CHWs and longer engagement may enhance acceptability.

A1c, Hemoglobin A1c; BP, Blood pressure; HLWD, Healthy Living with Diabetes; CHW, Community health worker.

a Used a 5-point Likert scale (1 = Very comfortable, 2 = somewhat comfortable, 3 = neutral, 4 = somewhat burdensome, 5 = very burdensome).

b High intervention adherence was defined as the attendance rate of group session > 80%; low intervention adherence was defined as the attendance rate of group session < 80% and/or did not cover all 5 topics in the phone calls in the intervention group.

c Used a 5-point Likert scale (1 = Very Dissatisfied, 2 = Dissatisfied, 3 = Okay; 4 = Satisfied; 5 = Very Satisfied).

d Used a 5-point Likert scale (1 = Completely disagree, 2 = Disagree, 3 = Neutral agree nor disagree, 4 = Agree, 5 = Completely agree).

e Used a 5-point Likert scale (1 = Not at all useful, 2 = Slightly useful, 3 = Somewhat useful, 4 = Very useful, 5 = Extremely useful).

#### 3.4.1 Recruitment and retention capability

The quantitative data showed in-person word of mouth as more useful in reaching potential participants compared to passive recruitment methods. Focus group data also confirmed and expanded that building relationships with potential participants was key to recruitment. Participants suggested utilizing more interactive methods and incorporating advanced multimedia through social media to promote the program for future recruitment.

Positive feedback from participants about the prerequisite orientation before informed consent may explain the high enrollment rate and acceptability to randomization. Qualitative data provided rich explanations on reasons for enrolling in the trial as it showed that the orientation helped them to understand study information and expectations and clarify their questions regarding participating in a trial. Participants also expressed no specific preference for trial groups because they wanted any kind of education, which may have resulted in no drop out after randomization. As for retention capability, conflicts with work schedules were reported as the main barriers for not staying in the program.

#### 3.4.2 Data collection acceptability

Although the process of collecting research data was reported as acceptable for participants based on survey data, qualitative interviews further indicated negative aspects. Participants suggested reducing the number of survey items to alleviate participants’ burden. Also, they suggested using electronic devices to collect survey data to enhance efficiency.

#### 3.4.3 Intervention adherence

Quantitative results showed that participants demonstrated high adherence to interventions and the qualitative findings further expanded the understanding of what made them adhere to the group sessions. Participants valued the weekly group sessions to keep them accountable in diabetes management. In the intervention group, peer support enhanced participants learning of new diabetes self-management skills as they received personal tips from their Ambassadors. One-on-one phone call discussions with the Ambassador were also recognized as reminders to participants of prior diabetes knowledge acquired during their group sessions, helping them to stay focused. Barriers to participation included schedule conflicts and significant life events.

When comparing the findings from participants with higher versus lower adherence to the intervention, several contributing factors were identified for future program improvement. For participants with low intervention adherence, long group sessions and difficulties in sharing thoughts related to diabetes self-management among other participants were reported. Additionally, they reported challenges in maintaining diabetes self-management practices, including finding it hard to break old habits (e.g., smoking) due to peer influence and adhering to medication. Participants expressed the need for someone to keep them accountable for the tasks they needed to do for managing diabetes. For the high adherence group, they faced different challenges in engaging with the program such as being hesitant to share their individual experience in front of their peers. The high adherence group tended to report the use of supplemental audio materials, which helped them complete the reading of the education materials each week.

#### 3.4.4 Intervention acceptability

Quantitative survey results showed participants were satisfied with the program. Participants also thought the program content and support they received was appropriate and useful for Black adults with type 2 diabetes. The qualitative findings further identified several key factors contributing to high program acceptability. For example, participants reported positive experiences with the intervention, resources, education, and safe space to share and learn from others’ disease experiences. The interactive nature of group sessions enhanced their self-efficacy for diabetes management because they were able to share their personal goals and receive individualized feedback to address barriers. Regarding the cultural relevancy of the group education session, participants preferred the dietary modifications discussed, which resonated with their cultural background and experience. However, some participants indicated less interaction during the group sessions and suggested incorporating videos or activities on exercise.

Participants also identified several points for future program improvement. Participants in the control group specifically suggested adding topics such as medication adherence, diabetes complications, and insurance information, and inviting healthcare professionals to the sessions. Regarding the support they received throughout the program, participants reported fewer opportunities to interact with the community health worker and recommended recruiting individuals with similar backgrounds and lived experiences.

## 4 Discussion

A culturally tailored diabetes management program that targets beliefs and provider mistrust and includes race-congruent peer support was feasible and acceptable for Black adults with type 2 diabetes. Results from our study showed recruitment should involve research teams engaging with potential participants in community events and allocating more time and resources explaining the rationale for the research and study expectations. Regular in-person group meetings with peer support and non-judgmental learning environments were key to engaging participants. Educational content that resonated with cultural experiences of the Black community and personalized action plans were reported to be essential for enhancing participants’ acceptability. However, barriers to participation, such as work schedule conflicts, repetitive survey questions, and lack of access to resources addressing social determinants of health, were also identified.

Our recruitment strategies were primarily based on our previous experience with a similar self-management program for Black adults with diabetes (22). The advantages of our recruitment approaches included well-established relationships with community partners embedded in the Black community as well as the leveraging the existence of advocates who enhanced word-of-mouth about the study. Of all recruitment methods, our results showed that community outreach was the most effective way to engage potential participants. The findings align with prior literature and our work that investigated the recruitment of African American adults for diabetes self-management programs, suggesting that in-person recruitment and snowball sampling may successfully reach research program participants (41–43). To continually build mutual collaborative relationships with Black individuals, we found that implementing motivational interviewing approaches along with sound health literacy practices in a study orientation, to explain research and address concerns related to health studies or the healthcare system in general, could be a promising way to enhance the enrollment and retention rates in marginalized populations (44).

Although study results indicated in-person recruitment was effective in approaching potential participants, creating multimedia promotional materials that incorporate elements such as personal experience and streaming on multiple social media platforms could be a more cost-effective channel (45). Another consideration for future recruitment is allocating resources to gatekeepers who can serve as contact persons in community organizations to collaborate with the study team. To the best of our knowledge, there is a lack of reference for setting recruitment goals for marginalized populations from the literature, including Black adults. In our study we revised our study recruitment goal because fewer participants were eligible. For future similar studies, we suggest a plan to reach out to three to four times the target sample size to enhance enrollment due to ineligibility.

Promoting accountability in self-management and providing flexibility for program participation were the core elements for adherence to the intervention. Our study participants identified regular group meetings with peer support as essential program components which helped maintain their long-term motivation for diabetes self-management. Consistent with prior studies using peer support in chronic disease management programs (46), our study further shows that race-congruent support enhances opportunities for learning self-management skills and also creates a safe environment to discuss structural discrimination issues Black adults experience in the healthcare system.

The study findings support attention to participants’ needs by providing alternative ways to study participation which could overcome barriers to program participation. In the post-pandemic era, diabetes self-management education and support programs recommendations include combining virtual options for education with in-person sessions to enhance adherence to interventions (47). Based on the recommendations from study participants, restructuring the module of program delivery could be the next step for enhancing accessibility for Black adults with diabetes who are in dire need of information about diabetes self-management and want to participate in such programs. Future studies could consider assessing the feasibility and acceptability of adapting to an online program, incorporating virtual group sessions, access to audiobooks and electronic data collection. Notably, few studies have strategically addressed barriers for program participants with lower intervention adherence. Our study results indicated that participants who are less adherent to intervention processes might need more time to open up and share their challenges in practicing self-management. They may prefer different interactive components during the delivery of an intervention to keep them focused during education sessions. This finding highlights the need for future studies to explore low-adherent participants’ perspectives on potential barriers and solutions for engaging in a diabetes self-management program.

This study underscores the critical role of culturally relevant interventions in promoting health equity and addressing diabetes disparities, particularly among Black adults. Both the quantitative and qualitative findings from this study showed that participants were satisfied with the group-based program with engaging discussion topics that were culturally tailored to Black adults. Leveraging the lived experience of two Black facilitators recruited directly from the community is aligned closely with literature which suggests that sharing cultural beliefs with Black adults helps foster pre-established trust (48). Additionally, the facilitators’ use of culturally appropriate language and discussion of self-management practices tailored to participants’ cultural backgrounds allowed the educational content to align with participants’ cultural and social contexts (49, 50). Despite this intentional cultural tailoring, some participants reported it was challenging for them to maintain a healthier lifestyle in the long term as they returned to neighborhoods that lacked exercise and where peer influences encourage bad habits. Unlike general diabetes self-management program services, our intervention specifically integrates key social determinants of health (SDOH), including race-concordant peer support, and strategies to address sociocultural barriers and medical mistrust which are relevant to Black adults. By embedding culturally relevant content, our findings align with evidence supporting the National Standards for DSMES, which emphasize the importance of addressing SDOH and leveraging culturally competent practices to ensure equitable access and engagement (51, 52). This approach not only enhances participant satisfaction and program effectiveness but also establishes a scalable model for delivering diabetes self-management education that bridges health equity gaps in diverse and marginalized populations. Future programs should consider expanding the scope of cultural adaptation to address social and environmental barriers to habit maintenance.

Regarding another behavioral aspect of diabetes self-management, our intervention showed a model for participants to develop self-advocacy skills to communicate with healthcare professionals. Participants reported a high perceived usefulness of provider-led group sessions where discussions about provider distrust and self-advocacy in asking questions were discussed. They reported they gained more confidence in interacting with their providers and asking more health-related questions. Utilizing healthcare professionals’ support to address concerns about disease and discuss beliefs about medication has been suggested in prior literature, especially for marginalized populations who may have mistrust of healthcare systems (53, 54).

In this intervention, we collaborated with a community health worker to address participants’ barriers related to social determinants of health such as transportation, childcare and housing, etc. However, participants did not use the resources as expected. Participant feedback showed less engagement with the support from the community health worker (CHW) due to limited time to interact with the CHW and lack of trust because the individual did not share similar lived experiences, including ethnic, racial and age backgrounds. To enhance participants’ acceptance of CHW support, race congruent CHW support could establish more robust connections with Black adults.

This study had several limitations. First, though this pilot study was not designed to test statistically significant outcomes, due to the recruitment challenges we faced, we were only able to implement the program with a relatively small sample size in one community setting in a Midwestern state in the United States. Although the study provides rich data on the feasibility of implementing a culturally tailored diabetes self-management program for Black adults with type 2 diabetes in a community setting, the findings may not apply to other settings because of geographical differences. Second, the scalability of this intervention was not a primary focus of this study; however, future research will explore adaptations to streamline delivery and evaluate cost and utilization outcomes to support broader implementation in larger-scale trials. Third, the study’s capacity only allowed us to recruit one community health worker to assist with participants’ social needs. Future studies should consider more participant engagement time community health workers in person and recruit those with shared backgrounds with participants.

## 5 Conclusion

A culturally tailored diabetes management program was found to be feasible to implement, highly acceptable and culturally appropriate for Black adults with uncontrolled type 2 diabetes. Findings from the mixed methods integration suggested that utilizing community outreach and leveraging multimedia advertisements were effective recruitment strategies. Although participants showed high adherence to the intervention and found group session content acceptable and useful, more effort is needed to enhance engagement for lower-adherent participants. Future work is required to explore the social and environmental barriers for Black adults to sustain healthy lifestyle changes after an intervention ends. A future large-scale trial that incorporates race-congruent support and addresses participation barriers is needed to evaluate for clinical and cost-effectiveness.

## Data Availability

The original contributions presented in the study are included in the article/Supplementary material, further inquiries can be directed to the corresponding author.
