# Supplementary material for: A pilot mixed methods randomized control trial investigating the feasibility and acceptability of a culturally tailored intervention focused on beliefs, mistrust and race-congruent peer support for Black adults with diabetes
Source: Front Public Health. 2025 Feb 6;13:1474027. doi: 10.3389/fpubh.2025.1474027 (PMC11841499; doi:10.3389/fpubh.2025.1474027)
Supplement: Supplementary file 1 [file Table_1.DOCX]

Supplementary Material

**Supplementary Figure 1.** Data collection joint display to summarize quantitative and qualitative research questions, data source and mixed methods integration by four feasibility domains.

| **Feasibility domain** | **Quantitative research questions and measures used** | **Qualitative research questions and data source** | **Mixed methods questions and  the reason for mixing methods*** | **Meta-inference** |
| --- | --- | --- | --- | --- |
| *Recruitment and retention capability* | 1. *Can we recruit our target population?* 2. *Where can we recruit our target population?*    1. Number of people expected to express interest       1. Target: 48 people       2. Data source: REDCap data    2. Number of participants who will be eligible and enrolled 3. Target: 24 participants 4. Data source: REDCap data    1. Number of participants recruited from various sources 5. Data source: REDCap data    1. Time from screening to enrollment 6. Target: 60 days 7. Data source: Dates on recruitment log and date consent form signed 8. *Can we keep participants in the study?*    1. Number of participants who remained in the study 9. Target: $\geq$80% retention rate 10. Data source: REDCap data | 1. *Where (and how) should we be recruiting?* 2. *How can we improve our recruitment pitch/materials?* 3. *What are the barriers and facilitators to study recruitment?* 4. *What life factors made participation difficult?* 5. *Reasons for participants who dropout* 6. Data source: Focus groups conducted with participants after the week 3 group session. 7. *What life factors made participation difficult?* 8. *Reasons participants dropout* 9. Field notes kept by study coordinator from calls with participants who dropped out; REDCap data | - - 1. *What recruitment strategies are feasible when the study recruitment rate and recruitment sources are combined with participants' recruitment experience and suggestions about the recruitment process?* **[Triangulation]**     2. *What recruitment facilitators and barriers are identified when the recruitment rate is combined with participants' experience of the recruitment process?* **[Completeness]**     3. *What individual or structural barriers to participating in the intervention are identified when retention rates are combined with participants' reasons for dropping out?* **[Completeness]** | - - - 1. Implications for adopting new recruitment strategies, partners, or locations.       2. Implications for improving the intervention including intervention components, material, mode of delivery and duration |
| *Randomization acceptability* | 1. *Can we randomize the target population?* 2. *Is randomization acceptable to potential participants?* 3. Number of enrolled participants who dropped out after randomization 4. Target: 20% drop-out rate 5. Data source: REDCap | 1. *Why did you decide to sign up for the study?* 2. *What did participants understand about the research rationale and study information?* 3. *What did participants understand about randomization?* 4. *How did it make you feel to be randomized in a study?* 5. Data source: Participant focus groups on week 3 session 6. *What are the reasons for declining to participate* 7. *Why did you withdraw after learning the group you were assigned to?* 8. Data source: Field notes taken by study coordinator | *What facilitators and barriers are identified when enrollment measures are combined with participants' experience of the consent and randomization process, influencing their decision to participate or decline?* **[Completeness]** | Implications for explaining research rationale, study information and randomization process to participants |
| *Data collection acceptability and adherence* | 1. *Are the assessments too burdensome?* 2. *Are the measures feasible and acceptable to participants?* 3. Participant satisfaction/burden ratings in Time 3 survey | 1. *How can we make data collection (A1C, survey, interview & focus group) easier?* 2. *Was anything in the surveys unclear?*    1. Data source: Participant interviews at the end | *What facilitators and barriers are identified when data assessment completion rate, satisfaction ratings, and duration of assessment visits are combined with participants' experience and suggestions regarding data collection?* **[Completeness]** | Implications for refining data collection, timing of data collection & revising outcomes |
| *Intervention acceptability* | 1. *Is the intervention acceptable to participants?* 2. Participant satisfaction ratings in Time 3 survey 3. Acceptability of Intervention Measure (AIM), Intervention Appropriateness Measure (IAM) and intervention component specific usefulness/helpfulness ratings in Time 3 survey | 1. *Feedback on group education session structure and facilitators/presenters* 2. Data source: Participant interviews after group sessions 3. *Most/least helpful aspects of group education content* 4. *Liked/did not like about intervention* 5. *Feedback on the role of CHW* 6. Data source: Participant interviews at the end | 1. *How do participant ratings of intervention acceptability compare to their satisfaction and perceptions of the intervention?* **[Triangulation]** 2. *What are intervention acceptability ratings between high/low intervention adherence groups? Why do ratings vary? What are participants' suggestions to enhance intervention acceptability?* **[Explanation]** | 1. Modifications to intervention components and materials 2. Compare qualitative responses between high/low intervention adherence groups to examine acceptability |
| *Intervention adherence* | 1. *Will participants do what they are asked to do?* 2. Weekly group session attendance    1. Target: $\geq$ 80% of sessions    2. Data source: sign-in sheets from group sessions in REDCap data 3. Phone call completion (intervention group only)    1. Target: $\geq$80% of phone calls    2. Data source: Field notes of weekly check-in calls with Ambassadors in REDCap data | 1. *What were the barriers to taking part in weekly group sessions?* 2. Data source: Participant interviews after group sessions 3. *What were the barriers to completing the phone calls with the ambassador? (intervention group only)* 4. Data source: Participants interviews at the end and Ambassador phone call documentation in REDCap data 5. *How do you include diabetes self-management practices into daily life?* 6. Data source: Time 3 interviews | 1. *What facilitators and barriers to adhering to the intervention are identified when intervention completion rates are combined with participants' experience of the intervention?* **[Completeness]** 2. *What factors hinder or facilitate adherence to the intervention between high/low intervention adherence groups?* **[Explanation]** 3. *How do participants' experience including diabetes self-management practices into daily life differ between high/low intervention adherence groups?* **[Explanation]** | 1. Modifications for mode of delivery and intervention duration 2. Compare qualitative responses between high/low intervention adherence groups to enhance intervention adherence groups |
